# Fucoxanthin@Polyvinylpyrrolidone Nanoparticles Promoted Oxidative Stress-Induced Cell Death in Caco-2 Human Colon Cancer Cells

**DOI:** 10.3390/md19020092

**Published:** 2021-02-05

**Authors:** Yue Sui, Yue Gu, Yujing Lu, Chenxu Yu, Jie Zheng, Hang Qi

**Affiliations:** 1 National Engineering Research Center of Seafood, Liaoning Provincial Aquatic Products Deep Processing Technology Research Center, School of Food Science and Technology, Dalian Polytechnic University, Dalian 116034, China; suiyuesydb1997@163.com (Y.S.); gu990713@163.com (Y.G.); lu990829@163.com (Y.L.); 2 Department of Agricultural and Biosystems Engineering, Iowa State University, Ames, IA 50011, USA; chenxuyu@iastate.edu; 3 Liaoning Ocean and Fisheries Science Research Institute, Dalian 116023, China

**Keywords:** fucoxanthin, nanoparticles, Caco-2 cells, pro-oxidation, targeted cell death

## Abstract

Fucoxanthin (FX), a natural carotenoid found in seaweed with multiple functional activities, is unstable with a poor water solubility that limits its utilization. This study aimed to improve FX’s stability and bioavailability via the nano-encapsulation of FX in polyvinylpyrrolidone (PVP)-coated FX@PVP nanoparticles (NPs). The FX@PVP NPs were evaluated in terms of their morphology, stability, encapsulation efficiency (EE), loading capacity (LC), and in vitro release to optimize the encapsulation parameters, and a 1:8 FX:PVP ratio was found to perform the best with the highest EE (85.50 ± 0.19%) and LC (10.68 ± 0.15%) and improved FX stability. In addition, the FX@PVP NPs were shown to effectively deliver FX into Caco-2 cancer cells, and the accumulation of FX in these cancer cells showed pro-oxidative activities to ameliorate H_2_O_2_-induced damage and cell death. The FX@PVP NPs could potentially become a new therapeutical approach for targeted cancer treatment.

## 1. Introduction

**Figure 1:**
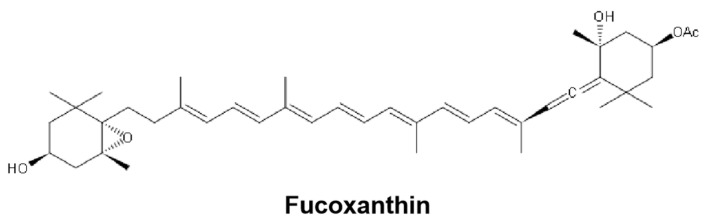
Chemical structure of fucoxanthin (FX).

Fucoxanthin (FX) is an organic pigment or carotenoid found in the photosynthetic organs of edible brown seaweeds [1]. The chemical structure of FX is shown in Figure 1. This compound has a unique structure, characterized by an unusual allenic bond, a conjugated carbonyl group, an epoxide group, and an acetyl group [2]. FX is a lipid-soluble pigment which widely exists in algae, marine phytoplankton, shellfish, and other animal and plant tissues [3]. FX has been shown to have many biological activities, such as anti-obesity [4], anti-cancer [5], anti-diabetes [6], and anti-oxidation [7] effects. The antioxidant activities of FX were confirmed in various cell-free systems, including the scavenging of hydroxyl radicals, superoxide radicals, singlet oxygen, DPPH radicals, 12-doxyl-stearic acid (12DS), and nitrobenzene with linoleic acid (NB-L) scavenging activity [8]. Moreover, FX inhibited ultraviolet B (UVB)-mediated oxidative damage to human fibroblasts [9] and hydrogen peroxide (H_2_O_2_)-mediated damage to monkey kidney fibroblasts (Vero line) [10]. However, as the flip side of an antioxidant, FX was also shown to behave as a pro-oxidant at certain concentrations which could reduce the cell viability [11]. This pro-oxidative role of FX increased the intracellular reactive oxygen species (ROS) production and extracellular signal-regulated kinase (ERK) 1/2 and p38 MAPK protein phosphorylation in murine hepatic BNL CL.2 cells [12], which potentially could be utilized to induce targeted cell death as a cancer treatment.

As a potential food supplement, FX has a good biocompatibility and biodegradability [13]. However, the structure of FX makes it susceptible to heat, light, oxygen, pH, enzymes, etc. [14]. In addition, its poor water solubility results in low bioavailability, which also limits the utilization of FX [15]. Thus, a critical need exists to improve the bioavailability and stability of FX.

Encapsulation is widely used to protect active substances and improve their physical and chemical properties as well as bioavailability [16]. Polyvinylpyrrolidone (PVP) is the most common water-soluble encapsulation carrier used in the preparation of solid dispersions. PVP dissolves well in water and many non-aqueous solvents due to the highly polar amide group within the PVP ring and apolar methylene and methine groups in the ring and along its backbone [17]. PVP, an amorphous polymer, can form complexes with other substances. The biocompatible and nontoxic nature of PVP renders it suitable for biotechnological applications [18]. PVP has also been widely used as stabilizer for nanoparticles (NPs) to prevent NP aggregation via the repulsive force arising from its hydrophobic carbon chains extending into the solvents to induce steric hindrance between NPs [19]. Nonetheless, to effectively use PVP for the nanoencapsulation of FX, the characterization of the actual improvement of the biological activity of FX due to encapsulation is much needed.

The objectives of this work were two-fold: (1) to encapsulate FX in PVP-passivated FX@PVP NPs to improve the solubility and stability of FX and (2) to characterize the effects of FX and FX@PVP NPs on H_2_O_2_-induced cell damage and cell death in Caco-2 cells. This study would provide a useful method for the development of an effective delivery system for bioactive compounds using nanoencapsulation for potential applications in functional foods, cosmetics, and therapeutics.

## 2. Results and Discussion

### 2.1. FX@PVP NPs Characterization

**Figure 2:**
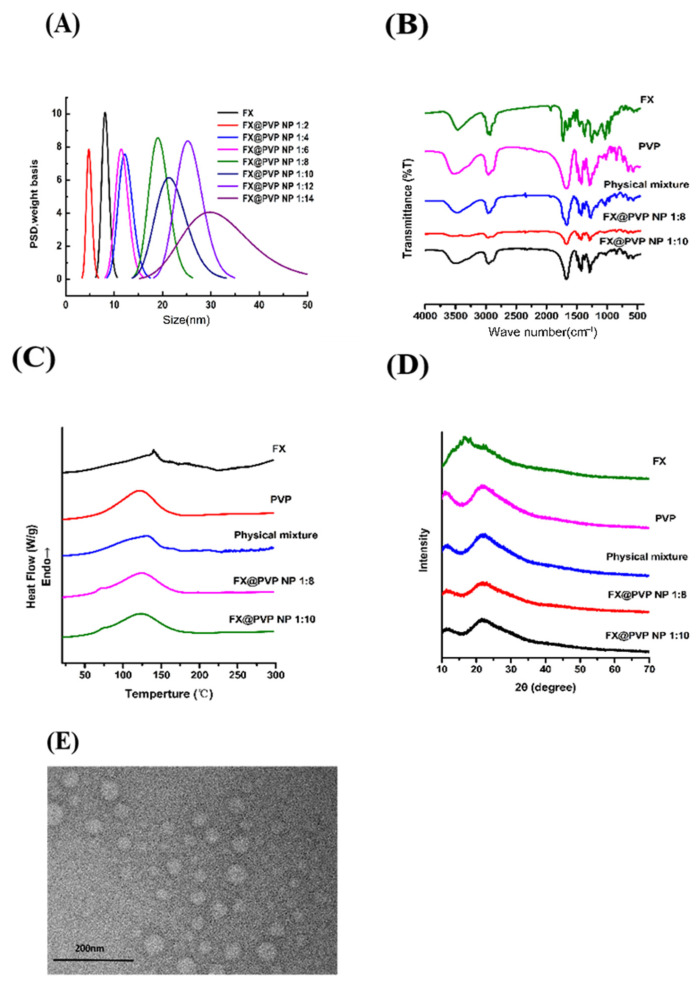
(**A**) Particle size of FX and FX@PVP NPs; (**B**) FTIR spectra of FX, PVP, physical mixture, and FX@PVP NPs; (**C**) DSC thermograms of FX, PVP, physical mixture, and FX@PVP NPs; (**D**) XRD patterns of FX, PVP, physical mixture, and FX@PVP NPs; (**E**) TEM images of FX@PVP NP 1:8.

Figure 2A shows the size distributions for FX@PVP NPs at different mass proportions. The NPs are uniformly dispersed in the ethanol solution and their sizes (diameter) are all less than 50 nm. With the increase in the content of PVP, the size of the formed NPs tended to enlarge.

The FTIR spectra of FX, PVP, physical mixture, and FX@PVP NPs are shown in Figure 2B to illustrate the binding between FX and PVP molecules. The specific bond groups identified in FX are as follows: the peak at 3295 cm^−1^: O–H stretching vibration; the peak at 1930 cm^−1^: the allenic bond (C=C=C), which is considered a functional group of FX; the peaks at 1714 and 1630 cm^−1^: C=O stretching; the peak at 1458 cm^−1^: C–H scissoring; the peaks at 1378, 1351, and 1334 cm^−1^: CH_2_ stretching; the peaks at 1078 and 1053 cm^−1^: –C–O stretching; and the peaks at 967 cm^−1^: C=C stretching. A carbonyl absorption band from PVP was detected at 1668 cm^−1^, which agreed well with the report of Koo et al. [20]. The spectrum of the physical mixture showed the absorption peaks of the two pure compounds, which indicated that no encapsulation between FX and PVP formed and that FX existed independently of PVP. In comparison, no characteristic FX peak was detected in the FX@PVP NPs spectra; most notably missing was the peak at 1930 cm^−1^, suggesting that FX was trapped inside the NPs probably through hydrogen bonding and became “invisible” to the spectrometer [21].

DSC thermograms of the samples are presented in Figure 2C. A sharp endothermic peak was observed for FX at around 140 °C, which was usually related to the degradation of chemical structures. In the PVP curve, there was no melting peak and a wide endothermic band at 75 to 150 °C was observed, which was associated with the loss of moisture, since PVP is a highly hygroscopic material [21]. The thermogram of the physical mixture showed the endothermic peaks of both PVP and FX, but no endothermic peak of FX could be seen in the DSC curve of the FX@PVP NPs. This strongly suggested that FX was encapsulated and that it might be amorphous in the FX@PVP NPs. This result was consistent with the FTIR results which were previously discussed.

The XRD patterns are shown in Figure 2D. The sharp and intense diffraction peaks of pure FX indicated that FX was in crystalline form. The XRD pattern of PVP showed no crystalline peaks. This confirmed that PVP was an amorphous polymer. FX peaks were observed in the physical mixture pattern, while the FX diffraction peaks in the NPs decreased with the increase in PVP content. The results indicated that FX gradually changes from a crystalline state to an amorphous state during the encapsulation process [22].

The transmission electron microscopy images of FX@PVP NP 1:8 are presented in Figure 2E. The NPs exhibited a uniform spherical shape and the NPs sizes were all less than 50 nm. This result was in agreement with the studies of Silva et al. [22] and Dos Santos et al. [21] reporting that PVP-encapsulated lutein and curcumin nanoparticles were spherical in shape.

### 2.2. Evaluation of the Encapsulation Efficiency (EE) and Loading Capacity (LC)

The EE and LC of the FX@PVP NPs samples are reported in Table 1. It was found that there was a significant difference between samples with different ratios (*p* < 0.05). FX@PVP NP 1:8 had the best results, with the EE at 85.50 ± 0.19% and the LC at 10.68 ± 0.15%. The difference in EE at different ratios may be caused by the degree of hydrogen bonding between FX and PVP. Nonetheless, since the best performance for the encapsulation was obtained at FX@PVP NP 1:8, these were the samples used in all subsequent investigations.

### 2.3. Stability of FX@PVP NPs

FX can easily degrade into other substances [23]. In the present study, the chemical stability of the FX in its encapsulated state was determined by measuring the content of FX in the samples subjected to various treatments.

**Figure 3:**
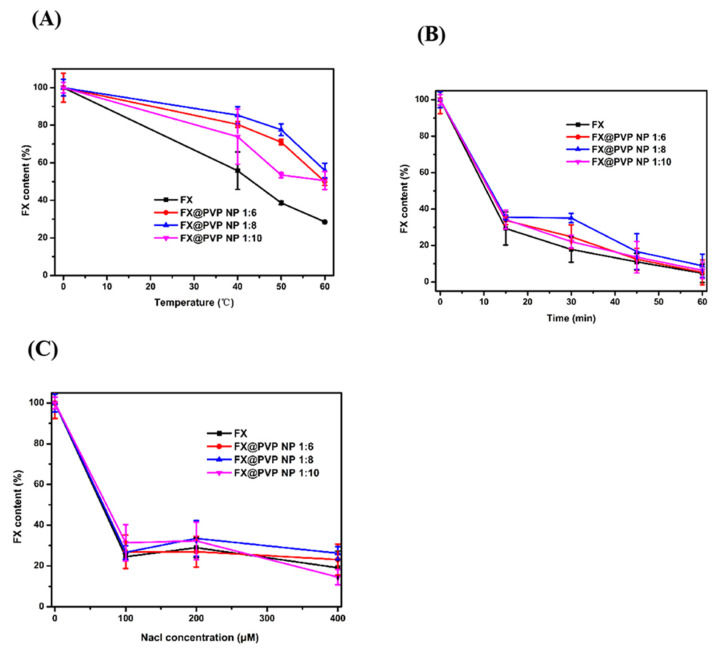
(**A**) Relative FX contents of FX and FX@PVP NPs heated at various temperatures up to 24 h; (**B**) relative FX contents of FX and FX@PVP NPs under UVB irradiation; (**C**) relative FX contents of FX and FX@PVP NPs prepared with different concentrations of NaCl.

The thermal stability results of FX and FX@PVP NPs are shown in Figure 3A. Under heating at 40 °C for 24 h, the content of FX in FX@PVP NP 1:8 was 85.39 ± 4.48%. This content was higher than that of free FX. With the temperature increased, the FX content in the samples decreased at a faster rate. This is consistent with reports from Huang et al. [24] and Sun et al. [25], which showed that free FX was unstable at temperatures above 40 °C. Zhao et al. [26] also reported that heating (from 25 to 60 °C) significantly promoted the degradation of FX. The FX content in FX@PVP NPs at 60 °C for 24 h was similar to that of free FX at 40 °C for 24 h, indicating that encapsulation did provide protection to FX against heat-induced degradation and could effectively improve the retention rate of FX with improved thermal stability.

The photo-stability results of FX and FX@PVP NPs are shown in Figure 3B. When the samples were irradiated under UVB for 15 min, the FX content decreased rapidly. As the irradiation time increased, the FX content of the samples decreased, and less than 10% remained after 60 min of irradiation. This is consistent with a previous study reported by Tavares et al. [27] stating that FX was unstable under UV irradiation. Regardless of the irradiation time, the FX content of FX@PVP NPs was always higher than that of free FX, indicating that the photo-stability of FX could be improved by encapsulation. The FX@PVP NP 1:8 appeared to perform the best, which could be attributed to its high encapsulation efficiency.

The stability of samples in salt solution for the FX and FX@PVP NPs are shown in Figure 3C. With the increase in NaCl concentration, the FX content flatten out after a quick initial drop. It appeared that PVP coating did not provide strong protection against salt for the FX. The FX@PVP NP 1:8 performed only slightly better than that of free FX in terms of salt stability.

**Figure 4:**
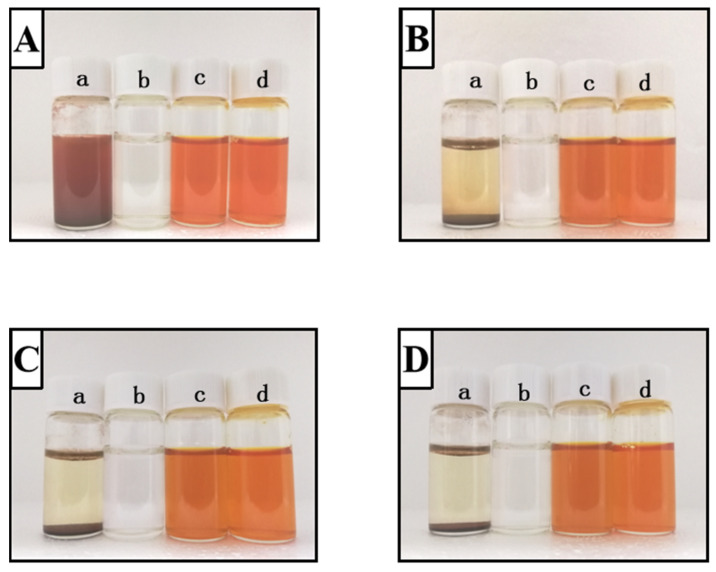
Photos of FX, PVP, and FX@PVP NPs solutions stored for different lengths of time. Images are representative of samples and depict storing for 0 day (**A**); 4 days (**B**); 8 days (**C**); 12 days (**D**). Key: (a) FX, (b) PVP, (c) FX@PVP NP 1:8, (d) FX@PVP NP 1:10.

The storage stability of FX, PVP, and FX@PVP NPs was assessed by observing the color changes of each sample stored at room temperature for 12 days (Figure 4). FX was insoluble in water and formed a red-brown suspension that was prone to precipitation. As the storage time increased, FX gradually settled, and after 12 days of storage the FX almost completely settled. PVP was completely dissolved in water, the solution was colorless and transparent with good stability, and no obvious changes occurred during storage. The FX@PVP NPs solution showed a uniform orange color at the beginning, and the color became slightly lighter as the storage time increased. Nonetheless, no precipitation was observed for the FX@PVP NP 1:8 sample, which showed good stability during storage. The color of FX @ PVP NP 1:10 was slightly lighter than that of FX @ PVP NP 1:8 after 8 days, and the difference became clear after 12 days of storage. The results indicated that PVP encapsulation could improve the storage stability of FX, and FX@PVP NP 1:8 had the best storage stability at room temperature when stored away from light.

### 2.4. In Vitro Release

**Figure 5:**
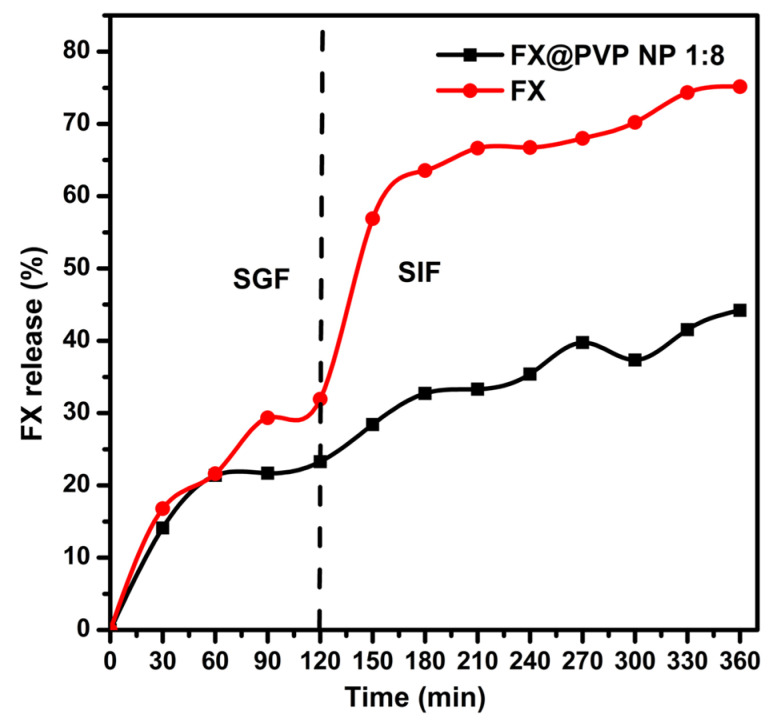
FX release profile in simulated gastrointestinal fluids.

FX and FX@PVP NP 1:8 were used for an in vitro digestion assay to evaluate the digestion stability and bioavailability of FX before and after encapsulation (Figure 5). In this assay system, the release of the FX from each sample was evaluated under pH conditions simulating the stomach (pH 2.1) and small intestine (pH 7.5), for 2 h and 4 h at 37 °C in the dark, respectively. For the un-encapsulated FX, the initial release in the simulated gastric fluid (SGF) was mild and reached 31.93 ± 0.13% at the 2 h mark. However, a burst of FX release occurred at the beginning of the simulated intestinal fluid (SIF) stage, similar to the experimental results reported by Oliyaei et al. [28]. After 4 h of the SIF stage, the release of FX reached 74.33 ± 0.14%. In comparison, for the FX@PVP NP 1:8 the release of FX was at 23.27 ± 0.15% at the end of the SGF stage, and a gradual yet slow release that continued into the SIF stage reached 44.22 ± 0.12% at the end of the SIF stage. The initial FX release from the FX@PVP NP 1:8 could be due to the rapid diffusion of FX located near the surface of the nanoparticles, comparable to the un-encapsulated FX, within 30 min. Once this stage has passed, the release of FX from the FX@PVP became steadier, possibly due to the much-slower diffusion of FX through the PVP shell. The results indicated that FX@PVP NPs could be effective to slow down the release of FX to sustain a well-controlled delivery of FX over the digestion process.

### 2.5. Effects of FX and FX@PVP NPs on Cell Viability

**Figure 6:**
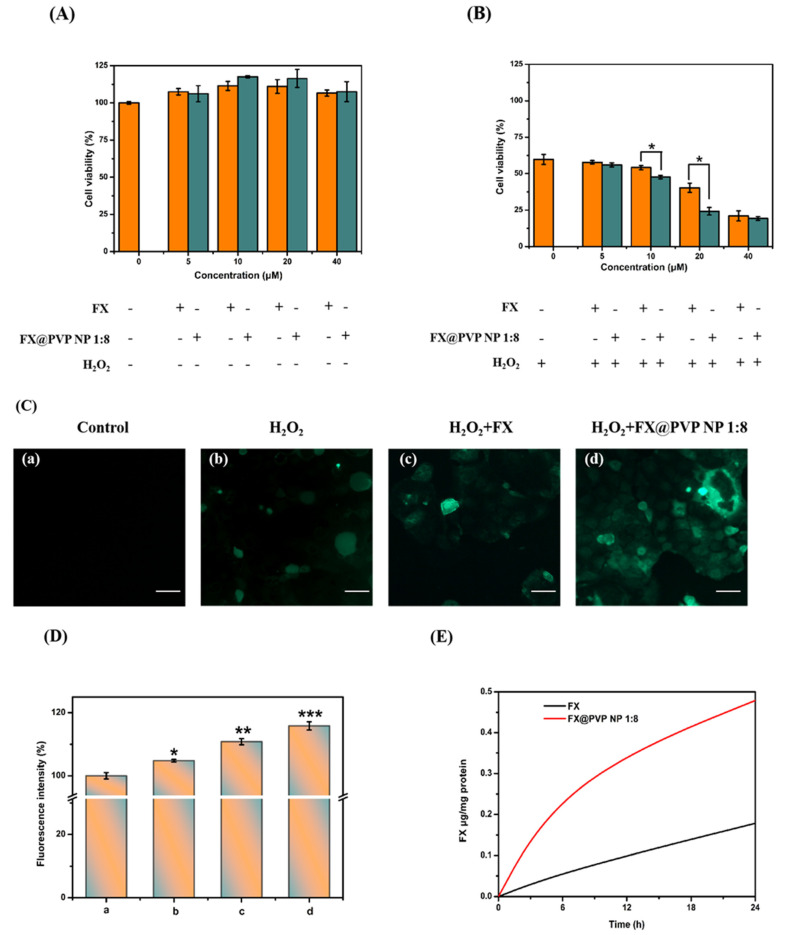
(**A**) Cell viabilities of FX and FX@PVP NPs against Caco-2 cells were incubated with different concentrations of FX and FX@PVP NPs and treatment with H_2_O_2_ (500 μM) for 24 h. (**B**) Cytotoxicity of Caco-2 treated with FX@PVP NPs at different concentrations without H_2_O_2_. (**C**) Fluorescent images of the treated cells were measured by using an inverted fluorescence microscope in H_2_O_2_-induced Caco-2 cells. Scale bars are 50 μm. (**a**) Caco-2 cells without treatment; (**b**) Caco-2 cell treatment with H_2_O_2_; (**c**) Caco-2 cells pretreated with FX (20 μM) prior to treatment H_2_O_2_; (**d**) Caco-2 cells pretreated with FX@PVP NPs (20 μM) prior to treatment H_2_O_2_; (**D**) Relative fluorescence intensity for (a–d); (**E**) The accumulation of FX and FX@PVP NP 1:8 in Caco-2 cells. Results are presented as mean ± SD; *n* = 4. * *p* < 0.05; ** *p* < 0.01; *** *p* < 0.001 compared to the control.

The cell viability MTT assay was conducted with Caco-2 treated with FX and FX@PVP NP 1:8 samples at different concentrations. As shown in Figure 6A, the viability of cells treated with both samples were higher than 100% regardless of concentration, indicating that FX and FX@PVP NP 1:8 were non-toxic to Caco-2 cells under normal conditions. Figure 6B showed the cell viability of Caco-2 treated with different concentrations of FX and FX@PVP NP 1:8 after H_2_O_2_ treatment. After the cells were pretreated with 500 μM of H_2_O_2_ for 24 h, the cell viability of the FX sample-treated groups was significantly reduced in a dose-dependent manner, and it dropped to about 20% at 40 μM (for both FX and FX@PVP groups). For the FX concentrations of 10 and 20 μM, the viability of Caco-2 cells treated with FX@PVP NP 1:8 was significantly lower than that of Caco-2 cells treated with the un-encapsulated FX. This might be due to the delivery of FX via FX@PVP NPs into Caco-2 cells being much more efficient than that via un-encapsulated FX. Apparently, after H_2_O_2_ treatment, FX showed pro-oxidant activity in Caco-2 cells, which accelerated Caco-2 cells apoptosis. These results verified the report of Shin et al. that under high oxidative stress in cancer cells, carotenoids act primarily as pro-oxidants, leading to apoptosis of cancer cells. [29] Hence, carotenoids such as FX can potentially be used for cancer treatment to induce targeted cell death.

### 2.6. Evaluation of Intracellular ROS Levels Affected by FX/FX@PVP NPs

ROS is an important indicator for the evaluation of the cellular antioxidant activities and the underlining molecular mechanism. When ROS exceeds the normal level, oxidative stress would accumulate to damage the structural integrity of cells, which could lead to the loss of cell function and ultimately causes cell death [30]. ROS level was detected via the fluorescence probe H2DCFDA. As shown in Figure 6C,D, H_2_O_2_-induced oxidative stress promoted ROS generation which was evidenced by the fluorescence from H2DCFDA in Caco-2 cells. After adding FX and FX@PVP NP 1:8 to Caco-2 cells pretreated with H_2_O_2_, the fluorescence intensity further increased, indicating that the FX acted as a pro-oxidant to enhance oxidative stress, which led to more Caco-2 apoptosis triggered by ROS. At a concentration of 20 μM, the fluorescence intensity of ROS generated by Caco-2 cells pretreated with H_2_O_2_ in FX@PVP NP 1:8 was significantly higher than that of FX. It was shown that the FX@PVP NPs had the strongest pro-oxidative effect. This might be due to the effective delivery of FX into Caco-2 cells. This phenomenon was consistent with the results of the MTT assay discussed earlier.

Previous research has shown that carotenoids could behave either as antioxidants or as pro-oxidants depending on their environments [31]. Inside normal cells, carotenoids are usually powerful antioxidants that reduce oxidative stress in cells [32,33]. However, carotenoids can function as pro-oxidants at high concentration and under abnormal conditions such as low levels of endogenous enzymes and antioxidants, imbalance and high intracellular oxidative stress (common in cancer cells), and so on [29]. Compared with normal cells, malignant cells tend to have lower levels of antioxidant enzymes and endogenous antioxidants, which hinders the normal metabolism of free radicals, hence, higher levels of ROS are usually seen in the cells [34]. The increase in ROS at lower concentration will trigger the tumor-promoting signals, which makes ROS show pro-cancer effects in cancer cells. However, the increase in ROS at high concentrations can enhance the anti-tumor signals and trigger the apoptosis of cancer cells induced by oxidative stress, so that ROS exhibit anti-cancer effects in cancer cells [35]. It has been confirmed that some carotenoids exhibit pro-oxidative activity to trigger the apoptosis of cancer cells through ROS production as a key mechanism [29]. Under normal physiological conditions, carotenoids are involved in the reduction in ROS through various mechanisms, such as electron transfer and the abstraction of allylic hydrogen atoms [36]. For example, carotenoids could interact with lipid peroxyl radicals (LOO•) via electron transfer to be converted into carotenoid radical cations (CAR+•) [36]. However, in cancer cells low concentrations of endogenous antioxidant enzymes may hinder the CAR+• regeneration. In turn, CAR+• may exhibit a pro-oxidant effect instead by catalyzing and propagating free radical chain reactions to increase the level of ROS and damage cell proteins, lipids, and DNA, leading to cancer cell apoptosis [37].

### 2.7. Assessment of FX Content in Caco-2 Cells

The accumulation of FX and FX@PVP NP 1:8 in Caco-2 cells is shown in Figure 6E. The FX content in Caco-2 cells treated with FX@PVP NP 1:8 reached 0.48 ± 0.0049 μg/mg protein after 24 h, and that of Caco-2 cells treated with un-encapsulated FX was only 0.18 ± 0.084 μg/mg protein. Apparently, the delivery of FX via FX@PVP NPs into Caco-2 cells was much more efficient than via un-encapsulated FX. As a potential cancer treatment via promoting oxidative stress induced cell apoptosis, the FX@PVP NPs could be quite effective.

## 3. Materials and Methods

### 3.1. Reagents and Materials

FX (≥98%, HPLC) was obtained from Chengdu Desite Biotechnology Co., Ltd. (Chengdu, China). Polyvinylpyrrolidone (PVP) was purchased from Sigma-Aldrich (St. Louis, MO, USA). Minimum Essential Medium (MEM) and trypsin (0.25% with EDTA) were purchased from Gibco (Thermo Fisher, USA). Penicillin and streptomycin solution, and PBS were obtained from HyClone (Logan, UT, USA). Fetal bovine serum (FBS), South America origin was obtained from PAN-Seratech GmbH (Aidenbach, Germany). Dimethyl sulfoxide (DMSO, >99.8%, GC) was obtained from Shanghai Aladdin Biochemical Technology Co., Ltd. (Shanghai, China). 3-(4,5-dimethylthiazolyl-2)-2,5-diphenyl tetrazolium bromide (MTT) and DMSO (Cell culture grade) were purchased from Beijing Solarbio Science & Technology Co., Ltd. (Beijing, China). H2DCFDA was purchased from Shanghai Macklin Biochemical Technology Co., Ltd. (Shanghai, China). HPLC grade methanol was purchased from Spectrum Chemical Manufacturing Corp-China (Shanghai, China). Other chemicals were of analytical grade and were used without further purification.

### 3.2. Preparation of the FX-Loaded PVP Nanoparticles

FX@PVP NPs were prepared following the method by Silva et al. [22] and Dos Santos et al. [21] with minor modifications. Briefly, PVP was dissolved in FX ethanol solution under magnetic stirring until a translucent solution was produced. The solution was then sonicated for 15 min, under pulse condition of 30 s on and 10 s off (Sonics VCX150, 130 W, 1/8’ tip and AMPL 40%). Formulations were prepared with the following mass proportions of FX@PVP NPs: 1:0, 1:2, 1:4, 1:6, 1:6, 1:10, 1:12, and 1:14 (m:m). The samples produced were freeze-dried into powder for subsequent experiments.

### 3.3. FX@PVP NPs Characterization

A laser particle size potential analyzer (DT 1202, Dispersion Technology, Inc. Bedford Hills, NY, USA) was used to determine particle sizes of the NPs. Fourier Transform Infrared (FTIR; Perkin Elmer Spectrum two, CT, USA) spectra were acquired in potassium bromide pellets, with resolution of 1 cm^−1^ from 4000 to 450 cm^−1^, in order to identify chemical interactions between FX and PVP. Thermal properties of the nanoparticles were determined by Differential Scanning Calorimetry (DSC250, TA Instrument, USA). Samples were weighed (3 to 5 mg) in sealed aluminum pans under nitrogen flow (50 mL/min) and heated from 20 to 300 °C at 20 °C/min. X-ray diffraction analyses (XRD; XRD7000, Shimadzu, Tokyo, Japan) were carried out from 10° to 70° (2θ) at 5°/min, using Cu Kα radiation generated at 40 kV and 30 mA, to reveal changes in the crystalline structure of FX after the encapsulation process. The morphological characterization of the FX@PVP NPs was evaluated using Transmission Electron Microscopy (TEM; JEOL model JEM 2100, 200 kV, Tokyo, Japan). Diluted samples were dripped onto 300 mesh parlodium-coated copper grids and dried under room temperature, then stained with 2% uranium acetate for analyzing.

### 3.4. Evaluation of the Encapsulation Efficiency and Loading Capacity

The encapsulation efficiency (EE) and loading capacity (LC) of FX were determined according to the procedure described by Priscila Dayane [21]. An aliquot of sample dispersion (2 mL) was kept in a convection oven at 50 °C until solvent evaporation. After that, distilled water (4 mL) was added and maintained at 25 °C for 3 h. The obtained solution was filtered through a syringe filter (0.45 μm porosity) and freeze-dried (Scientz-10ND, ZheJiang, China). The sample was then dissolved in methanol and its concentration was determined by high performance liquid chromatography (HPLC, Shimadzu SPD 20A, Tokyo, Japan). Methanol was used as the mobile phase at 0.5 mL/min in a C18 column (4.6 × 250 mm and particle size of 5 µm, Cosmosil, Japan) at 40 °C. Detection was performed by a photodiode array ultraviolet visible light detector (PDA) at 450 nm. The amount of FX that passed through the filtration membrane was considered to remain stable in FX@PVP NPs, encapsulation efficiency (EE%) was calculated by Equation (1) where C_FNP_ represent content of FX in the FX@PVP NPs and C_FX_ represent FX content used for encapsulation, respectively.

EE (%) = C_FNP_/C_FX_ × 100. (1)

Loading capacity (LC%) was calculated by Equation (2), where m_E_ is the mass of FX trapped in the nanoparticles and M is the mass of the dried nanoparticles.

LC (%) = m_E_/M × 100. (2)

### 3.5. Stability of FX@PVP NPs

The method for evaluating the stability of FX and FX@PVP NPs was slightly modified according to Tie et al. [38] and Liu et al. [39]. The thermal stability was assessed by placing equal volumes (1 mL) of samples in a water bath at different temperatures (40, 50, 60 °C) for 24 h, then the contents of FX in freeze-dried samples were determined by HPLC. The photo-stability was determined as follows: equal volume (1 mL) of samples freshly prepared was placed in the culture dish and subject to irradiation for 15 min, 30 min, 45 min and 60 min with UVB (wavelength range: 300–320 nm; peak: 311 nm; with a UVB lamp). After the testing samples were lyophilized, the content of FX was determined by HPLC. The stability of samples in salt solution was tested by adding sodium chloride to the samples to various final concentrations of NaCl (0, 100, 200, 400 μM, respectively). After mixing and removing insoluble matter, the solution was collected and freeze-dried, and the FX content of which was then determined by HPLC.

The dried samples were dissolved in water and stored at room temperature for 12 days during which the color change was monitored to determine the storage stability of the samples. Photographs were taken every 4 days.

### 3.6. In Vitro re Lease

In vitro release experiments of FX and FX@PVP NPs were carried out in two simulated digestive fluids, 0.1 M HCl solution of pH 1.2 and pepsin (0.3% *w*/*v*) as a SGF and 0.1 M NaHCO_3_ buffer solution adjusted to pH 7.5 with pancreatin (0.1% *w*/*v*) as simulated intestinal fluid (SIF), according to Oliyaei et al. [28] with slight modifications. About 0.025 g of samples were immersed in 10 mL of SGF and was vortexed in the dark for 120 min (37 °C), then added to 10 mL of SIF and incubated under the same condition for 240 min. Sampling was taken every 30 min. The FX released was analyzed using HPLC at 450 nm, and calculated according to Equation (3) where M_0_ is the amount of initial FX, and M_t_ is the amount of FX remaining in the samples at a given incubation time.

Fractional released (%) = (M_0_ − M_t_)/M_0_ × 100. (3)

### 3.7. Cell Cultures and Treatments

The Caco-2 human colon cancer cells were obtained from the Cell Bank of TongPai Biological Technology Co., Ltd. (Shanghai, China). Cells were reserved in MEM and enhanced with 20% FBS and 1% antibiotics at 37 °C in a humidified incubator at 5% CO_2_, with fresh media replaced every 2–3 days. FX and FX@PVP NPs were prepared by dissolving in dimethyl sulfoxide (DMSO). For the various experiments, samples were diluted to the desired concentrations using complete MEM.

The assessment of H_2_O_2_-induced cell damage was conducted according to Zheng et al. [40] with minor modifications. After medium was removed, the cells were treated with medium containing H_2_O_2_ (500 μM) for 24 h and incubated in culture medium with FX and FX@PVP NPs at different concentrations (0, 5, 10, 15, 20, and 40 μM) for 24 h. The Caco-2 cells were treated with vehicle control (DMSO).

### 3.8. Cell Viability Assay

The cell viability was evaluated by MTT assay with slight modifications [41]. Caco-2 cells were cultured in 96-well plates at 1.2 × 10^4^ cells per well and allowed to adhere for 24 h. Then, the cells were washed with fresh medium and treated. After 24 h of incubation, 10 μL of MTT (5 mg/mL) was added for 4 h at 37 °C. The medium were removed carefully, followed by the addition of 150 μL of DMSO to each well to dissolve the precipitate. The absorbance was then measured at 570 nm using a Tecan infinite 200 microplate reader (Tecan Trading AG, Switzerland). Cell viability was calculated by Equation (4) where A_t_ is the absorbance of treated cells and A_0_ is the absorbance of control.

Cell viability (%) = A_t_/A_0_ × 100. (4)

### 3.9. Evaluation of Generated Intracellular ROS Levels

The cellular ROS generation was observed by fluorescent inverted microscope (IX73, Olympus, Japan). Caco-2 cells (4 × 10^5^ cells/well) were seeded in 12-well plates and allowed to adhere for 24 h, followed by the treatment with H_2_O_2_ and various FX samples. Then, Caco-2 cells were collected via centrifugation and incubated in 1 mL fresh medium and 1 μL H2DCFDA (10 μM) at 37 °C for 30 min in the dark. Subsequently, the cells were rinsed with PBS three times. Fluorescent intensity of H2DCFDA was detected under inverted fluorescence Microscope which was proportional to cellular ROS generation [42]. ROS production was determined by a fluorescence microplate reader (Tecan Infinite 200 pro, Switzerland); the excitation wavelength was 485 nm and the emission wavelength was 535 nm. Relative fluorescence intensity was calculated by Equation (5) where FI_t_ is the fluorescence intensity of treated cells and FI_0_ is the fluorescence intensity of control.

Relative fluorescence intensity (%) = FI_t_/FI_0_ × 100. (5)

### 3.10. FX Content in Caco-2 Cells

In order to study the transport of FX and FX@PVP NPs into Caco-2 cells, a HPLC method was used. Briefly, Caco-2 cells were cultured in 6-well plates at 8 × 10^5^ cells per well and allowed to adhere for 24 h. After the medium was removed, the cells were again incubated in fresh culture medium with FX and FX@PVP NP 1:8 (40 μM) added for different time (0, 6, 24 h), the cells were then lysed and then lyophilized. Then the FX content was measured by HPLC, as previously described.

### 3.11. Statistical Analysis

All the measurements were conducted at least three times, and the experimental results were reported as means ± SD (standard deviation). The experimental results were analyzed by single factor analysis of variance (ANOVA) and Duncan’s multiple range tests using SPSS version 19 (SPSS, Chicago, IL, USA), and significant difference was considered to exist between the determined mean values when *p* < 0.05.

## 4. Conclusions

In the present study, a PVP encapsulation method was developed to create FX-loaded FX@PVP NPs for the better delivery and release control of FX. The best performing encapsulation results (loading efficiency of 85.50 ± 0.19%) were obtained at a FX:PVP ration of 1:8. The FX@PVP NPs provided protection for the FX to improve its stability as well as bioavailability. Both the MTT assay and the evaluation of the intracellular ROS levels showed that the FX@PVP NPs enhanced the transport of FX into Caco-2 cells, which was pro-oxidative in cells under oxidative stress and could induce more cell death. Potentially, the FX@PVP NPs could be used to promote ROS-triggered apoptosis in cancer cells to serve as a targeted cancer treatment.

## Figures and Tables

**Table 1:** Encapsulation efficiency and loading capacity of FX@PVP NPs.

FX@PVP NP(m:m)	Encapsulation Efficiency (%)	Loading Capacity (%)
1:2	64.75 ± 2.12 ^a^	32.37 ± 2.35 ^a^
1:4	26.36 ± 0.91 ^b^	6.59 ± 0.12 ^b^
1:6	21.49 ± 0.32 ^c^	3.58 ± 0.27 ^c^
1:8	85.50 ± 0.19 ^d^	10.68 ± 0.15 ^d^
1:10	82.61 ± 0.22 ^d^	8.26 ± 0.27 ^b^
1:12	84.89 ± 0.34 ^d^	7.07 ± 0.24 ^b^
1:14	69.15 ± 0.45 ^a^	9.68 ± 1.11 ^d^

Data are expressed as mean (±SD) (*n* = 7). Different letters indicate significant differences. *p* < 0.05 compared to the control.

## References

[1] Yan X., Chuda Y., Suzuki M., Nagata T. (1999). Fucoxanthin as the major antioxidant in Hijikia fusiformis, a common edible seaweed. Biosci. Biotechnol. Biochem..

[2] Hu T., Liu D., Chen Y., Wu J., Wang S. (2010). Antioxidant activity of sulfated polysaccharide fractions extracted from Undaria pinnitafida in vitro. Int. J. Biol. Macromol..

[3] Karpinski T.M., Adamczak A. (2019). Fucoxanthin-An Antibacterial Carotenoid. Antioxidants.

[4] Woo M.N., Jeon S.M., Shin Y.C., Lee M.K., Kang M.A., Choi M.S. (2009). Anti-obese property of fucoxanthin is partly mediated by altering lipid-regulating enzymes and uncoupling proteins of visceral adipose tissue in mice. Mol. Nutr. Food Res..

[5] Terasaki M., Kuramitsu Y., Kojoma M., Kim S.-Y., Tanaka T., Maeda H., Miyashita K., Kawagoe C., Kohno S., Mutoh M. (2020). High fucoxanthin wakame (Undaria pinnatifida) prevents tumor microenvironment formation in an AOM/DSS mouse carcinogenic model. J. Funct. Foods.

[6] Beppu F., Hosokawa M., Yim M.-J., Shinoda T., Miyashita K. (2013). Down-Regulation of Hepatic Stearoyl-CoA Desaturase-1 Expression by Fucoxanthin via Leptin Signaling in Diabetic/Obese KK-A y Mice. Lipids.

[7] Gammone M.A., Riccioni G., D’Orazio N. (2015). Marine Carotenoids against Oxidative Stress: Effects on Human Health. Marine Drugs.

[8] Sachindra N.M., Sato E., Maeda H., Hosokawa M., Niwano Y., Kohno M., Miyashita K. (2007). Radical scavenging and singlet oxygen quenching activity of marine carotenoid fucoxanthin and its metabolites. J. Agric. Food Chem..

[9] Heo S.J., Jeon Y.J. (2009). Protective effect of fucoxanthin isolated from Sargassum siliquastrum on UV-B induced cell damage. J. Photochem. Photobiol. B.

[10] Heo S.-J., Ko S.-C., Kang S.-M., Kang H.-S., Kim J.-P., Kim S.-H., Lee K.-W., Cho M.-G., Jeon Y.-J. (2008). Cytoprotective effect of fucoxanthin isolated from brown algae Sargassum siliquastrum against H_2_O_2_-induced cell damage. Eur. Food Res. Technol..

[11] Liu C.L., Huang Y.S., Hosokawa M., Miyashita K., Hu M.L. (2009). Inhibition of proliferation of a hepatoma cell line by fucoxanthin in relation to cell cycle arrest and enhanced gap junctional intercellular communication. Chem. Biol. Interact..

[12] Liu C.L., Chiu Y.T., Hu M.L. (2011). Fucoxanthin enhances HO-1 and NQO1 expression in murine hepatic BNL CL.2 cells through activation of the Nrf2/ARE system partially by its pro-oxidant activity. J. Agric. Food Chem..

[13] Li Y., Dou X., Pang J., Liang M., Feng C., Kong M., Liu Y., Cheng X., Wang Y., Chen X. (2019). Improvement of fucoxanthin oral efficacy via vehicles based on gum Arabic, gelatin and alginate hydrogel. J. Funct. Foods.

[14] Li H., Xu Y., Sun X., Wang S., Wang J., Zhu J., Wang D., Zhao L. (2018). Stability, bioactivity, and bioaccessibility of fucoxanthin in zein-caseinate composite nanoparticles fabricated at neutral pH by antisolvent precipitation. Food Hydrocoll..

[15] Zhu J., Sun X., Wang S., Xu Y., Wang D. (2017). Formation of nanocomplexes comprising whey proteins and fucoxanthin: Characterization, spectroscopic analysis, and molecular docking. Food Hydrocoll..

[16] Salvia-Trujillo L., Sun Q., Um B.H., Park Y., McClements D.J. (2015). In vitro and in vivo study of fucoxanthin bioavailability from nanoemulsion-based delivery systems: Impact of lipid carrier type. J. Funct. Foods.

[17] Koczkur K.M., Mourdikoudis S., Polavarapu L., Skrabalak S.E. (2015). Polyvinylpyrrolidone (PVP) in nanoparticle synthesis. Dalton Trans..

[18] Gupta B.S., Chen B.-R., Lee M.-J. (2015). Solvation consequences of polymer PVP with biological buffers MES, MOPS, and MOPSO in aqueous solutions. J. Chem. Thermodyn..

[19] You L.-P., Yan C.-H., Si R., Zhang Y.-W. (2006). Self-Organized Monolayer of Nanosized Ceria Colloids Stabilized by Poly (vinylpyrrolidone). J. Phys. Chem. B.

[20] Koo S.Y., Mok I.K., Pan C.H., Kim S.M. (2016). Preparation of Fucoxanthin-Loaded Nanoparticles Composed of Casein and Chitosan with Improved Fucoxanthin Bioavailability. J. Agric. Food Chem..

[21] Dos Santos P.D.F., Francisco C.R.L., Coqueiro A., Leimann F.V., Pinela J., Calhelha R.C., Porto Ineu R., Ferreira I., Bona E., Goncalves O.H. (2019). The nanoencapsulation of curcuminoids extracted from Curcuma longa L. and an evaluation of their cytotoxic, enzymatic, antioxidant and anti-inflammatory activities. Food Funct..

[22] do Prado Silva J.T., Geiss J.M.T., Oliveira S.M., Brum E.D.S., Sagae S.C., Becker D., Leimann F.V., Ineu R.P., Guerra G.P., Goncalves O.H. (2017). Nanoencapsulation of lutein and its effect on mice’s declarative memory. Mater. Sci. Eng. C Mater. Biol. Appl..

[23] Komba S., Kotake-Nara E., Tsuzuki W. (2018). Degradation of Fucoxanthin to Elucidate the Relationship between the Fucoxanthin Molecular Structure and Its Antiproliferative Effect on Caco-2 Cells. Mar. Drugs.

[24] Huang Z., Xu L., Zhu X., Hu J., Peng H., Zeng Z., Xiong H. (2017). Stability and Bioaccessibility of Fucoxanthin in Nanoemulsions Prepared from Pinolenic Acid-contained Structured Lipid. Int. J. Food Eng..

[25] Sun X., Xu Y., Zhao L., Yan H., Wang S., Wang D. (2018). The stability and bioaccessibility of fucoxanthin in spray-dried microcapsules based on various biopolymers. RSC Adv..

[26] Zhao D., Yu D., Kim M., Gu M.Y., Kim S.M., Pan C.H., Kim G.H., Chung D. (2019). Effects of temperature, light, and pH on the stability of fucoxanthin in an oil-in-water emulsion. Food Chem..

[27] Tavares R.S.N., Kawakami C.M., Pereira K.C., do Amaral G.T., Benevenuto C.G., Maria-Engler S.S., Colepicolo P., Debonsi H.M., Gaspar L.R. (2020). Fucoxanthin for Topical Administration, a Phototoxic vs. Photoprotective Potential in a Tiered Strategy Assessed by In Vitro Methods. Antioxidants.

[28] Oliyaei N., Moosavi-Nasab M., Tamaddon A.M., Fazaeli M. (2020). Encapsulation of fucoxanthin in binary matrices of porous starch and halloysite. Food Hydrocoll..

[29] Shin J., Song M.H., Oh J.W., Keum Y.S., Saini R.K. (2020). Pro-Oxidant Actions of Carotenoids in Triggering Apoptosis of Cancer Cells: A Review of Emerging Evidence. Antioxidants.

[30] Liu C., Guo H., DaSilva N.A., Li D., Zhang K., Wan Y., Gao X.-H., Chen H.-D., Seeram N.P., Ma H. (2019). Pomegranate (Punica granatum) phenolics ameliorate hydrogen peroxide-induced oxidative stress and cytotoxicity in human keratinocytes. J. Funct. Foods.

[31] Hosokawa M., Kudo M., Maeda H., Kohno H., Tanaka T., Miyashita K. (2004). Fucoxanthin induces apoptosis and enhances the antiproliferative effect of the PPARgamma ligand, troglitazone, on colon cancer cells. Biochim. Biophys. Acta.

[32] Arathi B.P., Sowmya P.R., Kuriakose G.C., Vijay K., Baskaran V., Jayabaskaran C., Lakshminarayana R. (2016). Enhanced cytotoxic and apoptosis inducing activity of lycopene oxidation products in different cancer cell lines. Food Chem. Toxicol..

[33] Gansukh E., Mya K.K., Jung M., Keum Y.S., Kim D.H., Saini R.K. (2019). Lutein derived from marigold (*Tagetes erecta*) petals triggers ROS generation and activates Bax and caspase-3 mediated apoptosis of human cervical carcinoma (HeLa) cells. Food Chem. Toxicol..

[34] Senthil K., Aranganathan S., Nalini N. (2004). Evidence of oxidative stress in the circulation of ovarian cancer patients. Clin. Chim. Acta.

[35] Reczek C.R., Chandel N.S. (2017). The Two Faces of Reactive Oxygen Species in Cancer. Annu. Rev. Cancer Biol..

[36] Ribeiro D., Freitas M., Silva A.M.S., Carvalho F., Fernandes E. (2018). Antioxidant and pro-oxidant activities of carotenoids and their oxidation products. Food Chem. Toxicol..

[37] Johnson J.D. (2009). Do carotenoids serve as transmembrane radical channels?. Free Radic. Biol. Med..

[38] Tie S., Zhang X., Wang H., Song Y., Tan M. (2020). Procyanidins-Loaded Complex Coacervates for Improved Stability by Self-Crosslinking and Calcium Ions Chelation. J. Agric. Food Chem..

[39] Liu C., Liu Z., Sun X., Zhang S., Wang S., Feng F., Wang D., Xu Y. (2018). Fabrication and Characterization of beta-Lactoglobulin-Based Nanocomplexes Composed of Chitosan Oligosaccharides as Vehicles for Delivery of Astaxanthin. J. Agric. Food Chem..

[40] Zheng J., Piao M.J., Keum Y.S., Kim H.S., Hyun J.W. (2013). Fucoxanthin Protects Cultured Human Keratinocytes against Oxidative Stress by Blocking Free Radicals and Inhibiting Apoptosis. Biomol. Ther..

[41] Rodriguez-Luna A., Avila-Roman J., Oliveira H., Motilva V., Talero E. (2019). Fucoxanthin and Rosmarinic Acid Combination Has Anti-Inflammatory Effects through Regulation of NLRP3 Inflammasome in UVB-Exposed HaCaT Keratinocytes. Mar. Drugs.

[42] Martínez S.R., Andrés M. (2021). Durantini, Revealing ROS Production by Antibiotics and Photosensitizers in Biofilms: A Fluorescence Microscopy Approach. Methods Mol. Biol..

